# Cost-Effective Thermomechanical Processing of Nanostructured Ferritic Alloys: Microstructure and Mechanical Properties Investigation †

**DOI:** 10.3390/ma17194763

**Published:** 2024-09-28

**Authors:** Yan-Ru Lin, Yajie Zhao, Yi-Feng Su, Thak Sang Byun

**Affiliations:** 1 Materials Science and Technology Division, Oak Ridge National Laboratory, Oak Ridge, TN 37830, USA; yzhao65@utk.edu (Y.Z.); suy1@ornl.gov (Y.-F.S.); 2 Department of Nuclear Engineering, University of Tennessee, Knoxville, TN 37996, USA

**Keywords:** oxide dispersion strengthened (ODS) alloys, nuclear materials, mechanical properties, nanostructured ferritic alloys, transmission electron microscopy (TEM), atom probe tomography (APT)

## Abstract

Nanostructured ferritic alloys (NFAs), such as oxide-dispersion strengthened (ODS) alloys, play a vital role in advanced fission and fusion reactors, offering superior properties when incorporating nanoparticles under irradiation. Despite their importance, the high cost of mass-producing NFAs through mechanical milling presents a challenge. This study delves into the microstructure-mechanical property correlations of three NFAs produced using a novel, cost-effective approach combining severe plastic deformation (SPD) with the continuous thermomechanical processing (CTMP) method. Analysis using scanning electron microscopy (SEM)-electron backscatter diffraction (EBSD) revealed nano-grain structures and phases, while scanning transmission electron microscopy (STEM)-energy dispersive X-ray spectroscopy (EDS) quantified the size and density of Ti-N, Y-O, and Cr-O fine particles. Atom probe tomography (APT) further confirmed the absence of finer Y-O particles and characterized the chemical composition of the particles, suggesting possible nitride dispersion strengthening. Correlation of microstructure and mechanical testing results revealed that CTMP alloys, despite having lower nanoparticle densities, exhibit strength and ductility comparable to mechanically milled ODS alloys, likely due to their fine grain structure. However, higher nanoparticle densities may be necessary to prevent cavity swelling under high-temperature irradiation and helium gas production. Further enhancements in uniform nanoparticle distribution and increased sink strength are recommended to mitigate cavity swelling, advancing their suitability for nuclear applications.

## 1. Introduction

Ferritic/martensitic (FM) steels are prime candidates for use in a range of advanced fission reactors and proposed fusion reactors [1,2,3], serving as cladding and structural materials [4,5], including those required for the first wall and blanket structures in fusion reactors [6,7]. In addition to FM steels, nanostructured ferritic alloys (NFAs) showed enhanced high-temperature tensile and creep properties [8,9,10]. Under irradiation, NFAs exhibited an extended operating temperature range compared to conventional FM steels and demonstrated superior resistance to cavity swelling [11,12]. These improvements were achieved by introducing a high density of nanoparticles, such as oxide-dispersion strengthened (ODS) steels [13,14], to increase the material sink strength [15,16]. NFAs are not limited to alloys containing oxide nanoparticles. Castable nanostructured alloys (CNAs) [17,18], which incorporate carbide or nitride nanoparticles, have also shown promising mechanical properties and radiation resistance. Despite advances in NFAs, current fabrication processes involving mechanical milling for mass production are costly and likely impractical due to their lengthy processing route. This study examined the microstructure and mechanical property correlation of three NFAs fabricated by a combination of low-cost severe plastic deformation (SPD) [19,20,21,22] and the continuous thermomechanical processing (CTMP) method [23,24]. The results demonstrated that our proposed fabrication process has the potential to produce desired nanostructured alloy properties, providing cost-effective manufacturing flexibility without significant scaling limitations.

Among all NFAs, 14YWT ODS alloys have been one of the standout materials specifically developed and undergoing refinement for nuclear applications [25,26,27]. The excellent high-temperature mechanical properties of 14YWT alloys, along with their radiation tolerance, are attributed to their ultra-small grains (100–300 nm), nanoparticle densities ranging from 10^23^ to 10^24^ m^−3^, and Y-Ti-O-enriched nanoparticle diameters as small as 2–10 nm [28,29,30]. Nevertheless, the exclusive method for producing high-quality 14YWT alloys is through high-power mechanical alloying (MA), involving multi-day ball milling of alloy powder mixed with yttria (Y_2_O_3_) powder, followed by consolidation and thermomechanical processing [31,32]. This complex pathway, particularly the low-temperature mechanical alloying, limits progress toward cost-effective production of 14YWT alloys. Hence, exploring and developing manufacturing techniques that bypass mechanical alloying yet achieve fine grains and high nanoparticle densities like those in 14YWT alloys will offer a significant advantage. Conversely, without a breakthrough solution, the significant benefits of nanoparticle dispersion in metallic materials may be absent from the future advancement of nuclear reactor technology.

Recently, Byun et al. proposed a low-cost SPD-CTMP method [33]. Using 14YWT base alloy powders (Fe-13.7Cr-2.9W-0.38Ti-(0.12–0.23)Y wt.%) produced via gas-atomization processes in a crucible, they applied a series of continuous thermomechanical processing cycles to impose high-temperature SPD conditions on the consolidated powder mixtures to produce 14YWT-based ODS alloys. They reported that alloys fabricated with the addition of Fe_2_O_3_ and Y_2_O_3_ to the 14YWT base alloy powders generally showed higher yield stress (YS) and ultimate tensile strength (UTS) compared to those made with only 14YWT base alloy powders. However, detailed microstructure characterization has not been conducted on these materials. This study selected three ODS alloys produced by the SPD-CTMP method with 14YWT base alloy powders only and the same powders with the addition of Fe_2_O_3_ or Y_2_O_3_ for further scanning electron microscopy (SEM), transmission electron microscopy (TEM), and atom-probe tomography (APT) characterizations to quantify the grain size and nanoparticle size and density. Results were compared to 14YWT alloys fabricated by the commonly used MA processes. This research provides valuable information for the optimization of ODS alloys produced by SPD-CTMP methods. The SPD-CTMP method could potentially be extended to a wide range of industrial applications—such as structural steels, pipes, and tubing—beyond nuclear reactors, where strength, durability, and cost-effectiveness are essential.

## 2. Materials and Methods

**Figure 1:**
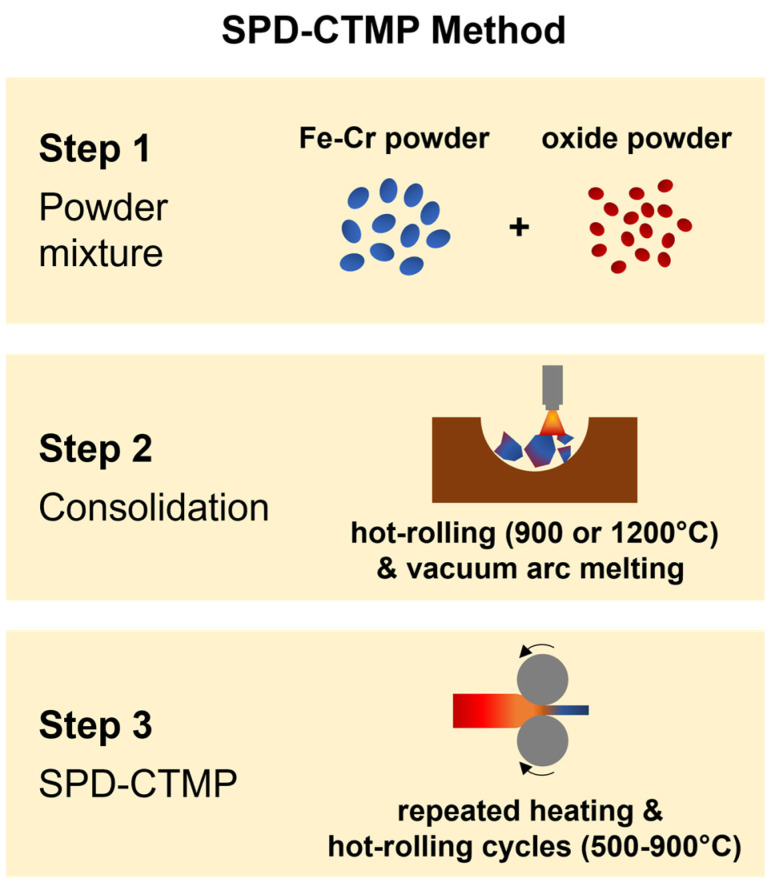
Schematic diagram of the alloy fabrication process using the SPD-CTMP method.

In this study, three different 14YWT-based ODS alloys (HR-1CC, HR-2CC, and HR-3A), produced using the SPD-CTMP method at Oak Ridge National Laboratory, were examined. Figure 1 illustrates the major steps and conditions for producing each material, including powder mixture, consolidation process, and CTMP. The primary difference among the three ODS alloys lies in the mixing powders (Table 1). Specifically, HR-1CC only used 14YWT-based powder alloyed with Y and O (Fe-14Cr-3W-0.4Ti-0.23Y-(0.07–0.14)O), HR-2CC included a mixture of 14YWT-based powder and Fe_2_O_3_, and HR-3A comprised a mixture of 14YWT-based powder and Y_2_O_3_. All compositions here are in weight percent (wt.%) unless noted otherwise. Further details of the material processing steps and conditions can be found elsewhere [23,33].

The tensile testing results for the three ODS alloys used in this study were obtained from our previous report [23,33]. Miniature SS-J2 type tensile specimens, measuring 5 mm × 1.2 mm × 0.5 mm and extracted from the central portion of HT-SPD processed plates using electrical discharge machining, were employed. Shoulder-loading cradle grips were used in TestResources and Instron systems for testing at room temperature and 500 °C, respectively. The tests were conducted at a nominal strain rate of 5 × 10^−4^ s^−1^, corresponding to a displacement rate of 0.15 mm/min for the 5 mm gauge section length of the SS-J2 specimen. Load-displacement data recorded during testing were used to determine engineering parameters, including YS, UTS, uniform elongation (UE), and total elongation (TE). Unless otherwise specified, tensile testing and data analysis were conducted in accordance with ASTM E8/E8M and E21 standards [34,35].

Microstructure characterizations were carried out on the same bulk samples used for tensile testing [33]. The initial step involved mounting and polishing one side of the samples to a colloidal silica finish using traditional metallography techniques. A TESCAN MIRA 3 SEM was employed for electron backscatter diffraction (EBSD) characterization to examine the microstructure and grain size of the fabricated ODS alloys. EBSD data were recorded using Oxford Aztec 6.1 software, with subsequent analysis performed with AZtecCrystal 3.1 software. Post-processing (data cleaning) included “auto-clean” (removes isolated single pixel errors and reduces low aggression data duplication) [36] and pseudo-symmetry removal steps for three sequences using the AZtecCrystal software. EBSD patterns were collected over surface regions of 45 μm × 30 μm (0.1 μm step size) for each sample to obtain a sufficient number of grains (minimum of 10 pixels per grain) for statistical analysis. The mean grain size is calculated as the average “equivalent circle size” of each grain, which represents the diameter of a circle with the same area as the grain. TEM samples (extracted from the grip area of the tensile specimens) were prepared via the focused ion-beam (FIB) method employing a Hitachi NB5000 SEM/FIB (Tokyo, Japan). The final thickness of the TEM-FIB samples was 80–100 nm, measured by the EELS log-ratio method with a measurement error of ±20% [37]. This error was accounted for in the error bars of the precipitate volume density. Characterizations employing scanning transmission electron microscopy (STEM) energy-dispersive X-ray spectroscopy (EDS) were performed using an FEI Talos F200X TEM (Hillsboro, OR, USA), operating at 200 kV, followed by analysis with FEI Velox 3.10 software. The size and density of the precipitates and nanoparticles were quantified using a self-developed deep learning model for automatic analysis [38]. APT data collection was conducted in laser mode using a CAMECA LEAP-4000X HR (Gennevilliers, France) with a detection efficiency of 36%. Data sets were gathered employing a base temperature of 50 K, a pulse rate of 200 kHz, a pulse energy of 50 pJ, and a detection rate ranging from 0.3% to 0.5%. The reconstruction of the acquired data was carried out using the Integrated Visualization and Analysis 3.6 Software (IVAS) developed by CAMECA. Consistently, the parameters utilized were: 33 V/nm evaporation field and a compression factor of 1.65. Reconstructions were calibrated with the interplanar spaces of <110> poles if they were apparent in the detector event histogram. In the cases where no clear pole was present, a field factor of 3.3 was used, which was close to the values obtained from the pole-based method.

## 3. Results

### 3.1. SEM-EBSD Characterization

**Figure 2:**
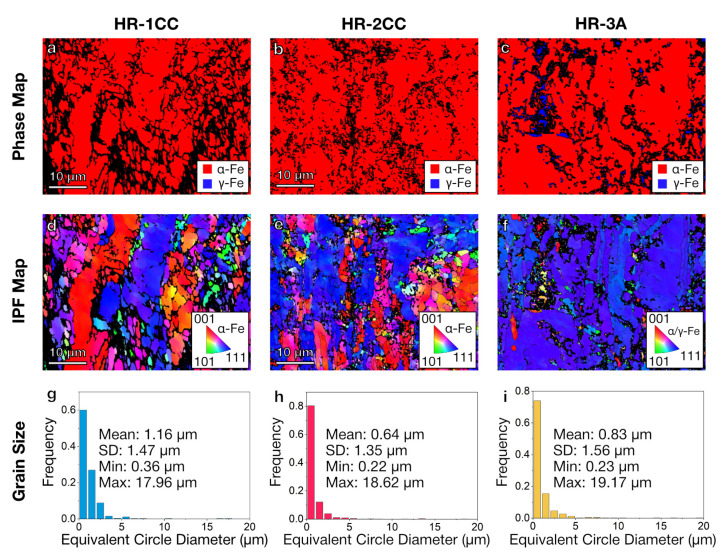
SEM-EBSD phase (**a**–**c**) and orientation maps and the grain structure (**d**–**f**) of HR-1CC (14YWT powder), HR-2CC (14YWT powder + Fe_2_O_3_), and HR-3A (14YWT powder + Y_2_O_3_) ODS alloys. (SD: standard deviation). (**g**–**i**) grain size distribution and statistics.

EBSD analysis was used to identify phases and examine the grain size distribution of the three 14YWT-based ODS alloys produced by CTMP methods (Figure 2). The phase map showed a dominance of α-iron (ferrite) with a body-centered cubic (bcc) crystal structure in all three alloys. Additionally, face-centered cubic (fcc) crystal structure was observed only in HR-3A, occupying ~17% of the surface area (Figure 2c). Although we used the γ-Fe (austenite) structure to fit the EBSD patterns, the fcc phase may actually be coarse precipitates with different compositions on the boundary, mistakenly identified as γ-Fe due to the constrained EBSD analysis. Additionally, considerable strain within the grains can induce enough distortion to misclassify them as the fcc phase when they are actually the bcc phase. In Figure 2d–f, the grains were color-coded based on the inverse pole figure (IPF) color map for an out-of-plane view (along the z-direction in the AZtec software) on the specimen. A preferred [100] and [111] orientation of the grains in the three alloys was indicated by the red and blue colors in the IPF map. The measured average grain sizes (equivalent circle diameter) for HR-1CC, HR-2CC, and HR-3A were 1.16 μm, 0.64 μm, and 0.83 μm, respectively. Although over 70% of the grains in the three materials fall within the range of 0.2–1 μm, a small number of larger grains measuring 17–19 μm were also observed.

### 3.2. STEM-EDS Characterization

**Figure 3:**
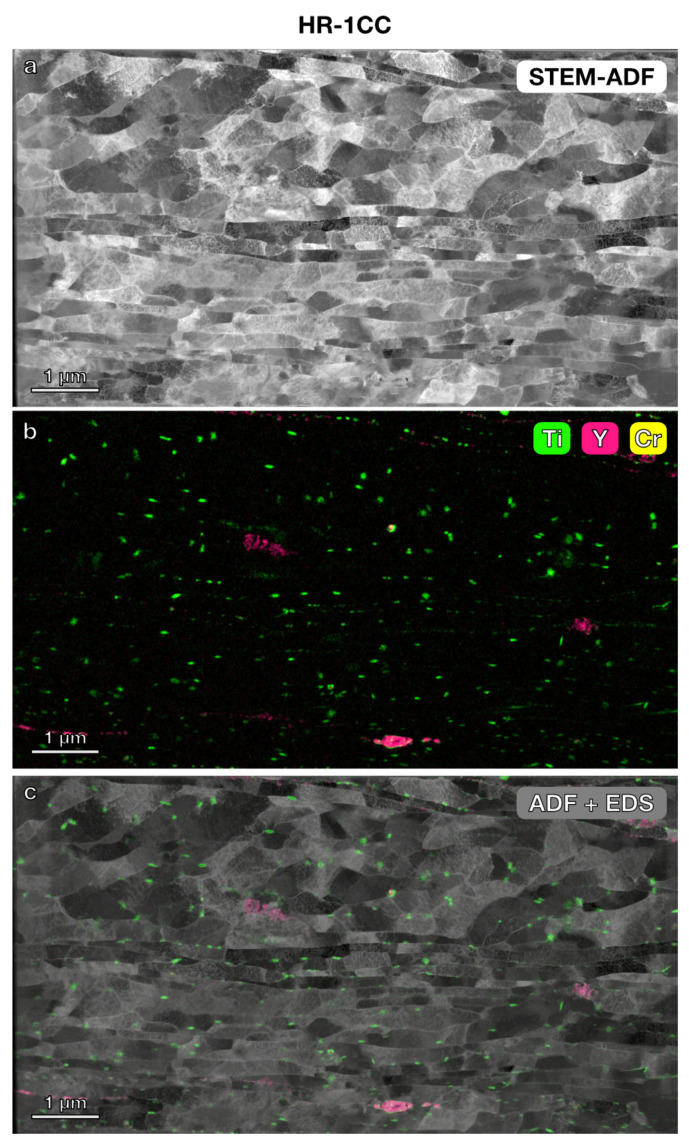
Microstructure and elemental mapping of HR-1CC (14YWT powder only): (**a**) STEM-ADF image, (**b**) STEM-EDS elemental map of Ti, Y, and Cr, (**c**) EDS map superimposed on STEM ADF image.

**Figure 4:**
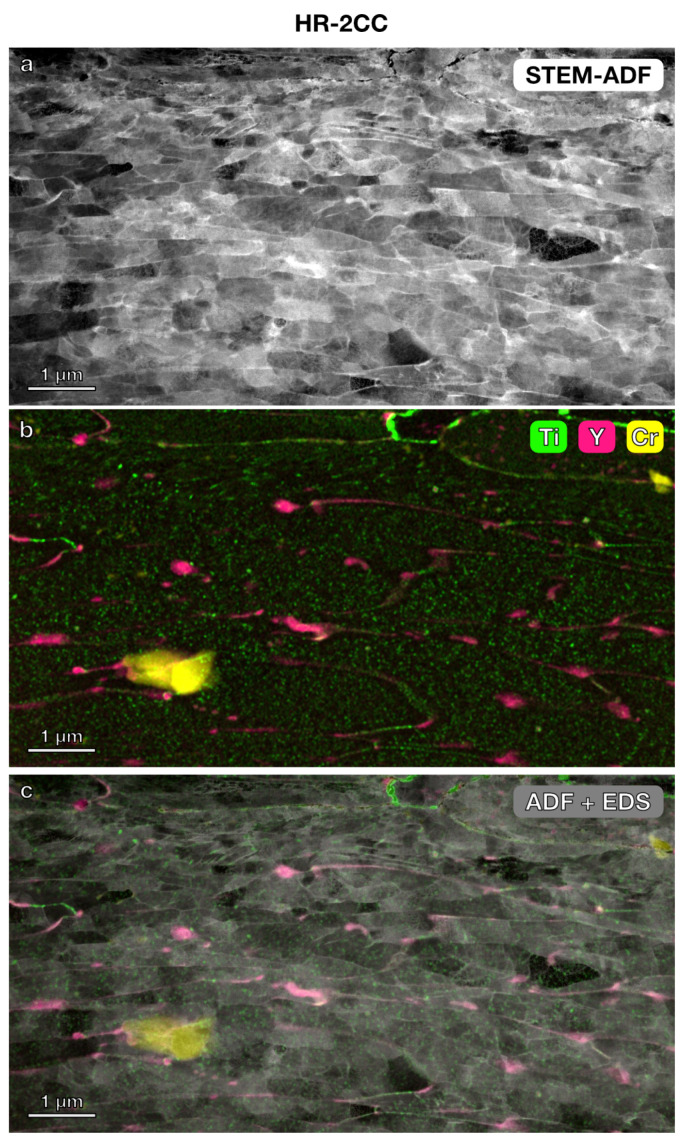
Microstructure and elemental mapping of HR-2CC (14YWT powder + Fe_2_O_3_): (**a**) STEM-ADF image, (**b**) STEM-EDS elemental map showing Ti, Y, and Cr, (**c**) EDS map superimposed on STEM ADF image.

**Figure 5:**
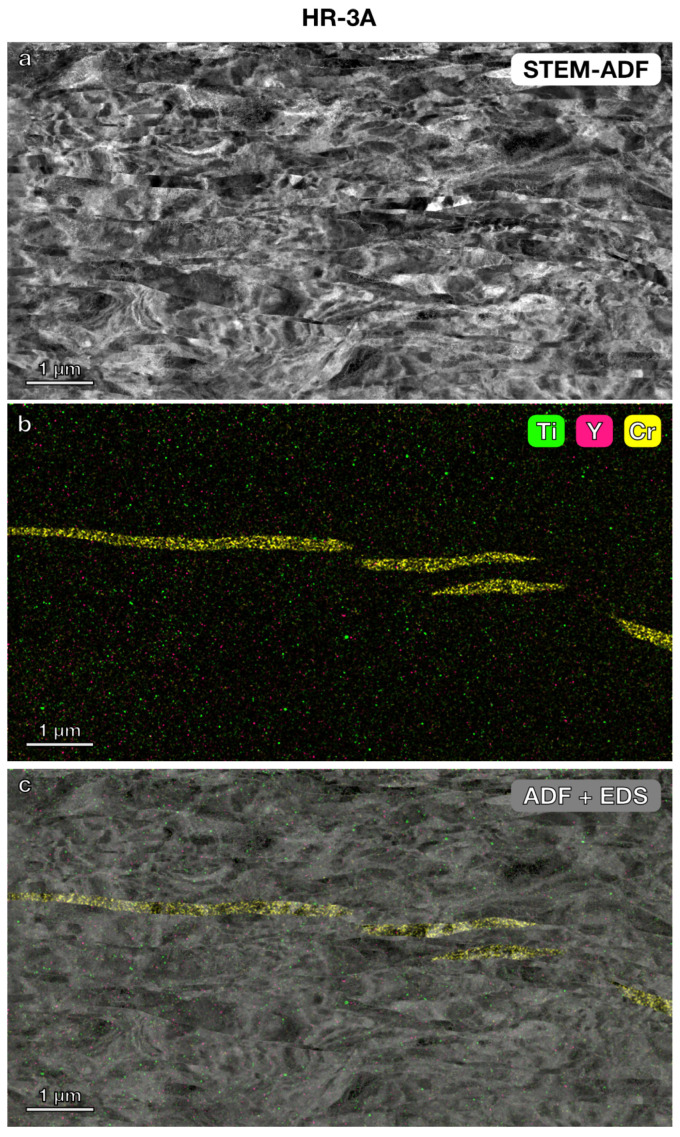
Microstructure and elemental mapping of HR-3A (14YWT powder + Y_2_O_3_): (**a**) STEM-ADF image, (**b**) STEM-EDS elemental map showing Ti, Y, and Cr, (**c**) EDS map superimposed on STEM ADF image.

**Figure A1:**
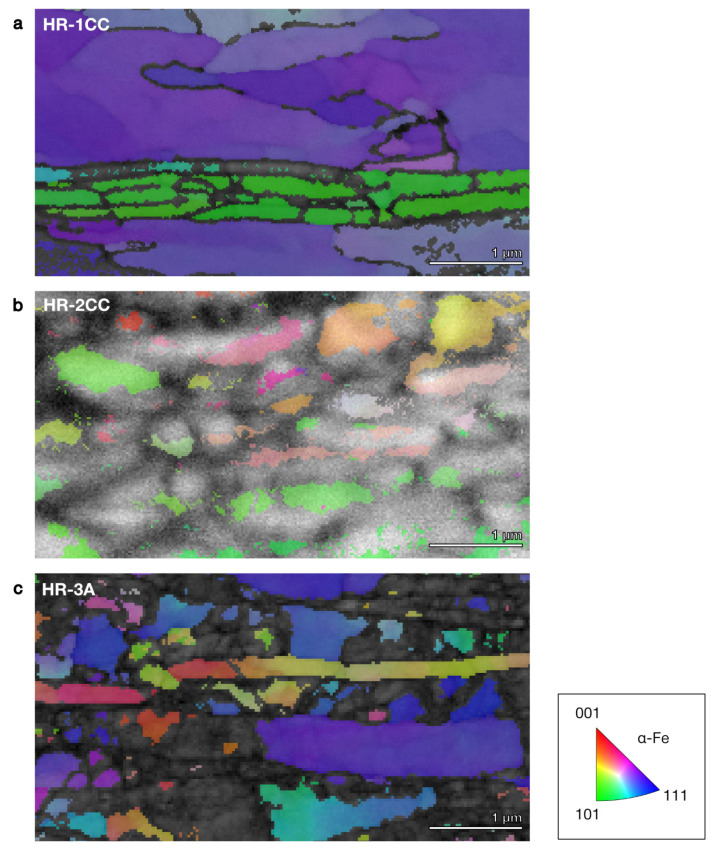
EBSD band contrast map overlaid with transmission Kikuchi diffraction (TKD) orientation maps obtained from the plan-view TEM sample of (**a**) HR-1CC, (**b**) HR-2CC, and (**c**) HR-3A.

STEM imaging and STEM-EDS elemental mapping were further used to investigate the microstructure and precipitates in the three nanostructured alloys. In the lower magnification STEM annular dark-field (ADF) images (Figure 3, Figure 4 and Figure 5) with a field of view of 6 μm × 4 μm, the grain boundaries were clearly visible. The average grain size measured using the average grain intercept (AGI) method [39] for the three samples ranged from 0.2 to 0.6 μm, which is consistent with the size of the majority of smaller grains (0.2–1 μm) measured by EBSD methods (Figure 2). Note that in this study, we assumed all boundaries were high-angle grain boundaries for ease of quantification. However, SEM-EBSD (Figure 2) and transmission Kikuchi diffraction (TKD) (Figure A1) analysis indicated that many of them are sub-grain boundaries. Ti-enriched clusters (in green) were observed in all three alloys in the EDS maps shown in Figure 3, Figure 4 and Figure 5. Y signals (in pink) were also detected in the EDS maps for these alloys. Larger Y-enriched clusters are clearly visible in HR-1CC (Figure 3) and HR-2CC (Figure 4). However, it’s uncertain whether the fine Y-enriched clusters in the EDS map of HR-3A (Figure 5) represent actual clusters or noise signals, particularly at lower magnifications. Large Cr-enriched clusters (in yellow) were observed in HR-2CC (~1 μm in size) and HR-3A (elongated strips measuring 2–4 μm in length) but were not observed in HR-1CC. The large Cr-enriched clusters in HR-3A may be correlated with the fcc phase observed in Figure 2c by the SEM-EBSD method. Additionally, grain-boundary enrichment of Y and Ti was observed in HR-2CC but not in the other materials (Figure 4). Ti and Y-enriched clusters in HR-2CC were unevenly distributed. Ti-enriched clusters were seen in grains with Y grain-boundary enrichment, while Y-enriched clusters were found in grains with Ti grain-boundary enrichment (Figure 4b).

**Figure 6:**
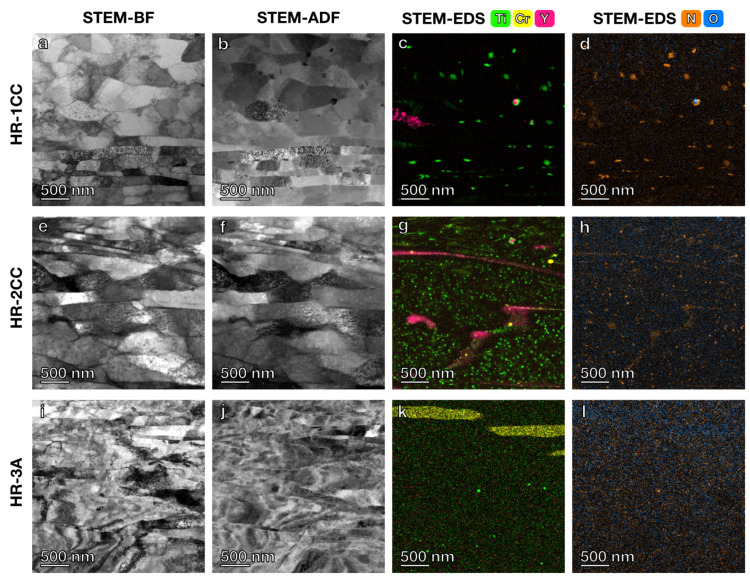
STEM BF and ADF images with corresponding EDS elemental maps for: (**a**–**d**) HR-1CC (14YWT powder only), (**e**–**h**) HR-2CC (14YWT powder + Fe_2_O_3_), and (**i**–**l**) HR-3A (14YWT powder + Y_2_O_3_) alloys.

Comparing the microstructures of the three alloys at a higher magnification with the field of views of 2 μm × 2 μm (Figure 6) revealed finer Ti, Y, or Cr-enriched clusters. Additionally, Ti-enrichment was observed at the interface of the Y-enriched clusters in HR-CC (Figure 6c) and HR-2CC (Figure 6g). Similar to Figure 5, it is uncertain whether the Y signals (in pink) in the EDS map of HR-3A (Figure 6k) are from real Y-enriched clusters with diameters <5 nm or simply noise. There was no overlap of Ti and Y signals in the EDS maps; Ti signals overlapped with N signals, while Y and Cr overlapped with O signals. Therefore, in the following section, we classified these fine clusters into three types: Ti-N, Y-O, and Cr-O.

**Figure 7:**
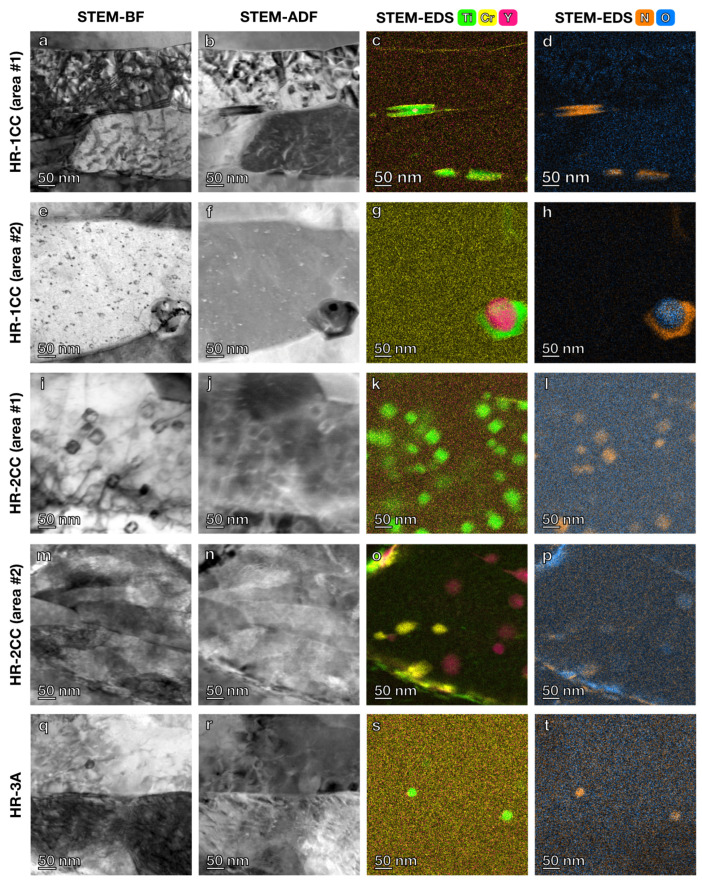
High-magnification STEM BF and ADF images with corresponding EDS elemental maps for Ti, Y, and Cr-enriched clusters in: (**a**–**h**) HR-1CC (14YWT powder), (**i**–**p**) HR-2CC (14YWT powder + Fe_2_O_3_), and (**q**–**t**) HR-3A (14YWT powder + Y_2_O_3_).

STEM-EDS maps at higher magnifications were used to examine the shape and local details of the precipitates and to determine if finer clusters exist in the three alloys (Figure 7). The black lines and dot features in the STEM bright-field (BF) images are likely dislocation lines or defect clusters caused by FIB damage (Figure 7a,e). As the Ti, Y, and Cr-enriched clusters were not homogeneously distributed, several different areas of the TEM-FIB sample were examined, each with a field of view of 300 nm. The projected shapes of the Ti-N clusters in HR-2CC (Figure 7k) and HR-3A (Figure 7s) were nearly square or circular, while those in HR-1CC (Figure 7c) were more oval or rectangular. The Ti-N clusters in HR-1CC predominantly formed at the grain boundaries. Additionally, Cr enrichment was observed at the grain boundaries and interfaces of the Ti-N clusters in HR-1CC (Figure 7c), and fine Cr-O clusters were only observed in HR-2CC (Figure 7o). The Y-O clusters were nearly circular in both HR-1CC (Figure 7g) and HR-2CC (Figure 7o). Fine Y-O clusters were not observed in HR-3A (Figure 7s), with higher magnification STEM-EDS maps.

**Figure 8:**
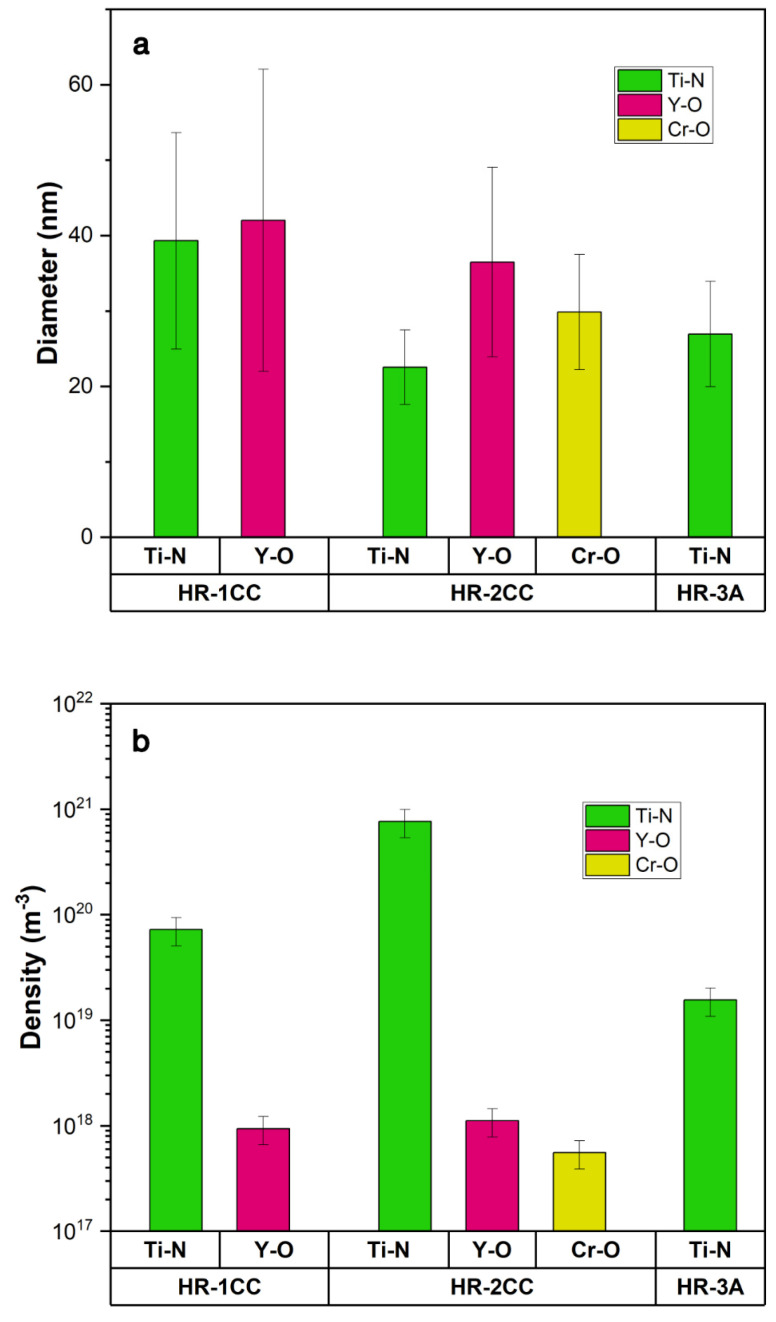
Comparison of the (**a**) size and (**b**) density of Ti, Y, and Cr-enriched clusters in HR-1CC, HR-2CC, and HR-3A alloys.

The size and density of the Ti-N, Y-O, and Cr-O clusters were quantified using STEM-EDS maps and are summarized in Figure 8. Overall, Ti-N clusters are the predominant type of cluster in the three alloys. HR-2CC had an order of magnitude higher density of Ti-N clusters (7.6 × 10^20^ m^−3^) compared to HR-1CC (7.2 × 10^19^ m^−3^) and HR-3A (1.5 × 10^19^ m^−3^) (Figure 8b). The average diameters of the Ti-N clusters in HR-1CC, HR-2CC, and HR-3A were 39.3 nm, 22.5 nm, and 26.9 nm, respectively (Figure 8a). The size and density of the Y-O clusters in HR-1CC and HR-2CC were comparable, at approximately 40 nm and 1 × 10^18^ m^−3^, respectively. Fine Cr-O clusters were observed exclusively in HR-2CC, with an average diameter of 29.8 nm and a density of 5 × 10^17^ m^−3^. In Figure 8a, considering the error in the average diameter, the size differences between the various precipitates may not be significant, generally falling within the range of 20–40 nm.

### 3.3. APT Characterization

**Figure 9:**
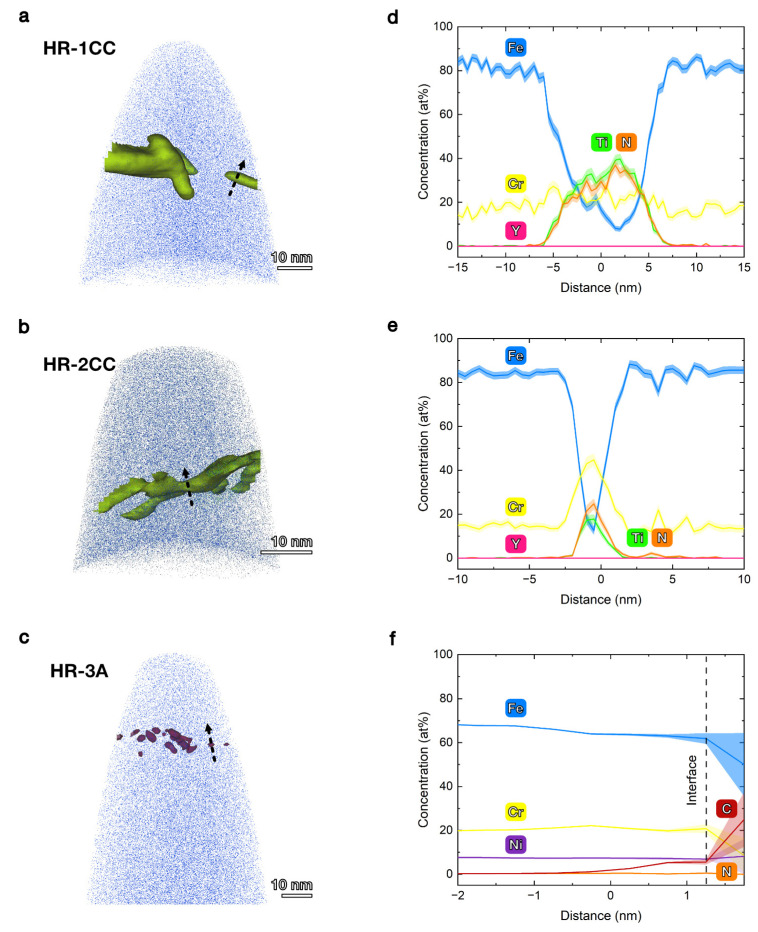
APT reconstruction of (**a**) HR-1CC, (**b**) HR-2CC, and (**c**) HR-3A CTMP alloys and the corresponding compositional profile (**d**–**f**) across the interfaces marked by the black arrows in (**a**–**c**).

**Figure A2:**
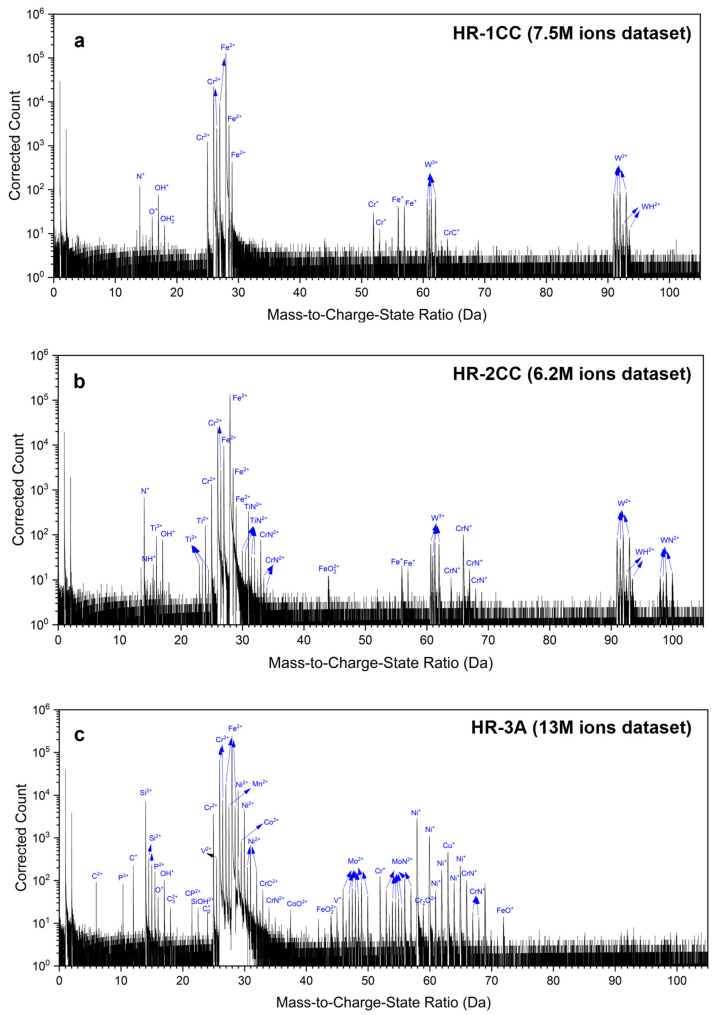
Mass spectrum from the APT analysis of (**a**) HR-1CC, (**b**) HR-2CC, and (**c**) HR-3A.

APT (Figure 9) was conducted to quantify the atomic concentration of elements in the nanoparticles and to determine if finer particles (<10 nm) formed in the HR-1CC, HR-2CC, and HR-3A alloys, which may not be detected by STEM-EDS methods (Figure 7). Except for HR-3A, where finer carbon-enriched clusters (likely carbides) with a diameter of ~2 nm were observed, no fine Y-O clusters were detected in the three alloys. No significant Y peaks were observed in the APT datasets of the three alloys (see Figure A2 for the APT mass spectrum). Consistent with the STEM-EDS results (Figure 7), the formation of nitrides in HR-1CC (Figure 9a) and HR-2CC (Figure 9b) was confirmed by the presence of TiN and CrN peaks in the mass spectrum. For the Ti-N particles in HR-1CC, both Ti and N concentrations were 30–40 at%, with no significant change in Cr concentration compared to the matrix (Figure 9d). For the N-enriched particles in HR-2CC, the Ti and N concentrations were 20–25 at%, with a peak Cr concentration of ~45 at% in the particles (Figure 9e). In addition to Fe (~70 at%) and Cr (~20 at%) in HR-1CC (Figure 9f), Ni (7.27 at%), Mn (1.34 at%), and Si (1.06 at%) were also detected, indicating possible impurities in the powder or those introduced during the manufacturing process. The decomposition of peak overlaps is based on natural abundance. Chemical compositions were measured from proximity histograms and 1-D concentration profiles.

## 4. Discussion

### 4.1. Correlation between Microstructure and Mechanical Properties

**Figure 10:**
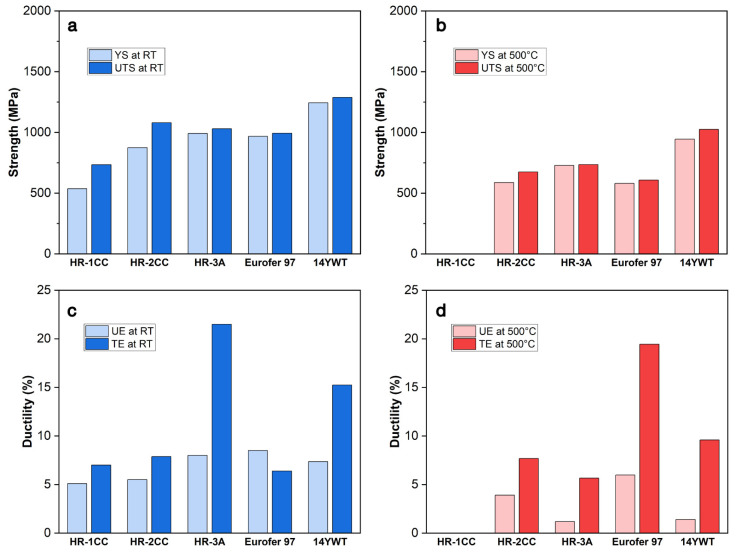
Comparison of yield strength (YS), ultimate tensile strength (UTS), uniform elongation (UE), and total elongation (TE) measured at (**a**,**c**) room temperature (RT) and (**b**,**d**) 500 °C for ODS-Eurofer 97, 14YWT-SM13 ODS alloys, and ODS alloys produced by the CTMP method (HR-1CC, HR-2CC, and HR-3A). Data from references [31,33,44].

Figure 10 presents a comparison of YS, UTS, UE, and TE for ODS alloys produced by the CTMP method in this study, alongside ODS-Eurofer 97 [40] and 14YWT-SM13 ODS alloys [41] produced via mechanical milling. These properties were measured at both room temperature and 500 °C. The production of ODS-Eurofer 97 alloys in Ref. [40] involved multiple steps, starting with the base alloy from Böhler, followed by inert gas atomization, and mechanical alloying with 0.3 wt.% Y_2_O_3_, hot isostatic pressing (HIP), and hot rolling by Plansee. The ODS-Eurofer 97 material was then normalized at 1100 °C, quenched, and tempered at 750 °C to achieve a tempered martensitic structure. Generally, the YS and UTS of the ODS FM alloys decrease with increasing test temperatures [33,42,43]. The comparison of YS and UTS in Figure 10a,b shows that the strength of mechanically milled 14YWT-SM13 is 1.2–2.3 times higher than that of the HR-series alloys produced by the CTMP method. However, the HR-series alloys exhibit strengths (in the range of 500–1000 MPa) comparable to, or slightly better than, ODS-Eurofer 97 alloys, which are also produced by mechanical milling. Among the three HR-series alloys, HR-2CC and HR-3A demonstrate notable room temperature strengths with YS > 800 MPa and UTS > 1 GPa, maintaining YS and UTS above 550 MPa at 500 °C.

Regarding UE and TE of ODS alloys, the ductility parameters exhibit complex temperature dependencies, strongly influenced by the processing route and the material’s strength [45,46]. Previous studies have reported that unique micromechanisms in FM steels, such as dynamic strain aging (DSA), can affect the strength of steels within certain temperature ranges [47,48]. In general, at room temperature, ODS alloys with higher strength parameters exhibit lower ductility parameters [49,50]. Byun et al. [33] reported that UE and TE of the HR-series alloys decreased with increasing test temperatures below 300 °C. However, between 300 and 500 °C, UE and TE of ODS alloys may increase with increasing temperature. In Figure 9c,d, the HR-3A alloy exhibited UE and TE values comparable to those of the highest strength material, 14YWT, at both room temperature and 500 °C. ODS-Eurofer 97 had the highest UE and TE values at 500 °C compared to other materials. The strength and ductility of ODS-Eurofer 97 were generally similar to the HR-series alloys at room temperature, except for the TE of HR-3A. Both our STEM-EDS and APT characterizations detected a small amount of Ni in HR-3A, which is likely related to the unusual TE and UE results and the formation of the γ-Fe (austenite) phase observed in EBSD maps (Figure 2c). It is possible that Ni, Mn, Si, or other impurities were introduced during the alloy processing for HR-3A.

The strength and ductility of ODS and other nanostructured alloys were suggested to be related to the size and density of nanoparticles and grain structures [51]. Table 2 summarizes the grain size, fine particle size, and density of 14YWT, ODS-Eurofer 97, and the HR-series alloys compared in Figure 10. The 14YWT alloy contained the highest density of Ti-Y-O particles, on the order of 10^23^-10^24^/m^3^, and the smallest average grain size (~200 nm). In contrast, the HR-series and ODS-Eurofer 97 alloys contain nanoparticles at much lower densities (10^19^-10^22^/m^3^) and have relatively coarser grain structures (0.6–3 µm). Although the ODS-Eurofer 97 and HR-series alloys showed comparable strength and ductility (Figure 10), the ODS-Eurofer 97 alloy had larger average grain sizes (2–4 times) compared to HR-series alloys. Additionally, the fine particle density in ODS-Eurofer 97 was 2–3 orders of magnitude higher than in HR-series alloys. This suggests that both average grain size and fine particle density influence mechanical properties, with grain size likely having a more pronounced effect. Additionally, although limited data is available, dislocation density might also be an influential factor in the strength and ductility of nanostructured alloys. It is unclear if the nano-grain structure produced via the low-cost, semi-annealed CTMP method would have a high density of dislocations sufficient to suppress grain size and nanoparticle effects. The mechanical performance of our trial material has not yet reached the level of well-established high-temperature materials like 14YWT ODS alloys produced by high-power mechanical alloying (involving ball milling). However, the results demonstrate the significant untapped potential of our approach for future alloy design. Most importantly, while the ball milling process can take up to a week, the CTMP process requires only 1–2 h. This significantly reduces both the time and cost of producing nanostructured alloys. Future efforts aimed at optimizing the mechanical properties of nanostructured alloys produced through CTMP methods should prioritize achieving a uniform distribution of grain structures and fine particles, reducing grain size, and enhancing fine particle density. Additionally, understanding cyclic fatigue damage is crucial. Further investigation into fatigue crack initiation and propagation is needed to examine the fatigue limit of these alloys [52].

### 4.2. Formation of Precipitates

The STEM-EDS (Figure 3, Figure 4 and Figure 5) and APT (Figure 9) results showed no large Fe_2_O_3_ and Y_2_O_3_ powders, indicating that the Fe_2_O_3_ and Y_2_O_3_ powders (initial powder size ~µm) mixed for HR-2CC and HR-3A were dissolved during the CTMP alloy processing. However, confirmed by the APT analysis, the dominant type of fine particles was Ti-N (with a density of 10^−19^–20^21^) m^−3^, rather than Y-O (with a density of ~10^−18^). This suggests that the HR-series alloys produced by the CTMP method in this study may primarily be nitride dispersion strengthened (NDS) rather than oxide dispersion strengthened. The CTMP material processing steps were conducted under an inert argon gas or vacuum environment. However, the results indicate that nitrogen, likely from impurities in the Fe-Cr-based metal or oxide powders, reacted with other elements in the powder, leading to the formation of nitrides. Alternatively, Springer et al. [53] used Ar in the laser synthesis process to lower the N_2_ partial pressure. They reported that despite the much lower Gibbs free energy of formation for oxides compared to nitrides, the higher N_2_ partial pressure in the arc process favors nitriding. Additionally, Smith et al. [54] observed stress-induced nitride formation when attempting to produce ODS alloys. The SPD step used for producing the HR-series alloys could introduce significant stress, which may have contributed to the formation of nitrides. Although the formation of nitrides was not expected, replacing oxides with nitrides or using a combination of both could offer various benefits. Mathon et al. [55] reported that NDS alloys exhibit remarkable ductility at high temperatures, in contrast to ODS alloys. This may explain why we observed better ductility in the HR series alloys compared to 14YWT (Figure 10c,d).

**Figure 11:**
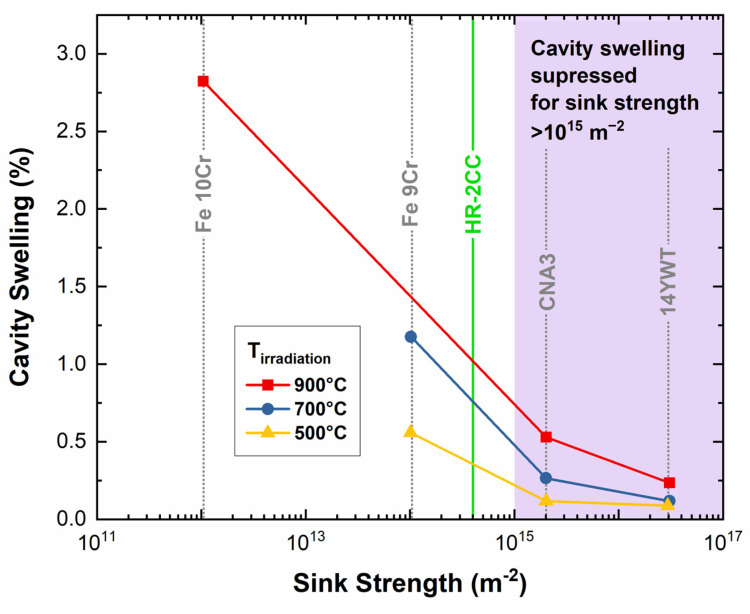
Variation of cavity swelling as a function of initial sink strength. (Figure reproduced from [28]). The purple-colored area in the figure represents the estimated sink strength of nanoparticles required to suppress cavity swelling.

Uniform dispersion of nitrides and oxides in alloys both exhibited improvement in radiation resistivity, such as cavity swelling commonly observed in materials under high-temperature irradiations. Lin et al. [28] reported that to suppress cavity formation by distributing the formation of transmutant He and providing high-density sink sites for point defect recovery, the initial sink strength should be above 10^15^ m^−2^. Assuming that grain size and fine nanoparticles in the HR-series alloys dominate the sink strength, the calculated sink strength of grain boundaries ($S_{gb}=60/d^{2},\;$where d is the average grain size) and nanoparticles ($S_{p}=4\pi rN$, where r is the particle radius and N is the particle density) for HR-2CC (which has the highest particle density of the HR-series alloys) was 2.2 × 10^14^ m^−2^ and 1.7 × 10^14^ m^−2^, respectively. As shown by the green line in Figure 11, the sum of the two values gives a total initial sink strength ($S_{gb}+S_{p}$) of 3.9 × 10^14^ m^−2^. This indicates that the HR-series alloys, although close to 10^15^ m^−2^ and better than Fe-Cr alloys without nanoparticles (Fe-9Cr and Fe10Cr in Figure 11), may not effectively suppress cavity swelling at higher irradiation temperatures with significant helium levels ~10,000 appm [28]. For fast fission reactor applications with a lower helium production rate of ~0.1 appm He/dpa, the required sink strength level for cavity suppression may be lower, around 10^14^ m^−2^, depending on the temperature and dpa level. In Figure 11, CNA3 is a castable nanostructured alloy [56] with the addition of fine nitrides or carbides, with a density and average diameter of 10^21^–10^22^ m^−3^ and 3–20 nm, respectively. Although larger particles and lower density may not be sufficient for swelling prevention, they may aid in high-temperature creep since nanoparticles are not effective dislocation obstacles and do not inhibit grain boundary migration [57]. Note that cavity swelling is linked to the ratio of biased to unbiased sink strengths [16,58]. Figure 11 calculations account only for nanoparticle density and size, but sink strength can vary dynamically under high-temperature neutron irradiation [59]. Additionally, Figure 11 assumes that the initial sink strength of dislocations is much lower than that of nanoparticles. However, in cases of high dislocation density (e.g., cold-worked alloys [60]), dislocations can surpass nanoparticles in sink strength, rendering the Figure 11 calculation invalid. As a result, high dislocation density from deformation can help reduce swelling [61]. To optimize nanostructured alloys produced through CTMP methods, it is necessary to further reduce grain size, improve homogeneity, increase the density of fine particles, and elucidate the mechanisms behind oxide and nitride formation. Since oxides, nitrides, and carbides are stable at various temperatures, adding different types or combinations of these precipitates may expand the application of NFAs depending on the operating temperatures and environments.

## 5. Conclusions

This study underscores the significant untapped potential of the SPD-CTMP method for future alloy design. This method is highly efficient, reducing production time from over a week with ball milling to just 1–2 h with the CTMP process, significantly cutting costs for nanostructured alloys. By examining microstructures in three NFAs produced via the CTMP method and comparing them with mechanically milled alloys like 14YWT and ODS-Eurofer 97, we observed notable relationships between microstructure and mechanical behavior. The HR-series alloys, despite lower nanoparticle densities compared to 14YWT ODS alloys, show competitive strengths and notable ductility, likely due to fine grains. However, increasing nanoparticle densities may be necessary to suppress cavity swelling at higher irradiation temperatures. Furthermore, our findings shed light on oxide and nitride formation in FM steels using the CTMP method. The unexpectedly high density of nitrides may result from nitrogen impurities in the powder or atmosphere, revealing more about the intricacies of alloy formation with this technique. Future efforts to optimize the mechanical properties and radiation resistance of nanostructured alloys through CTMP methods should prioritize achieving uniform grain and fine particle distribution, reducing grain size, enhancing particle density, and clarifying mechanisms behind oxide and nitride formation.

## Figures and Tables

**Table 1:** Material processing conditions for ODS alloys examined in this study.

Sample ID	Powder Mixture (wt.%)	Consolidation Process	SPD-CTMP Process
HR-1CC	Fe-13.7Cr-2.9W-0.38Ti-0.23Y-0.07O	6 hot-rolling cycles at 900 °C for 80% strain	8 hot-rolling cycles at 600 °C for 220% strain
HR-2CC	Fe-13.7Cr-2.9W-0.38Ti 0.23Y + (0.22)Fe_2_O_3_	6 hot-rolling cycles at 900 °C for 80% strain	8 hot-rolling cycles at 600 °C for 220% strain
HR-3A	Fe-13.7Cr-2.9W-0.38Ti-0.12Y + (0.3)Y_2_O_3_	6 hot-rolling cycles at 900 °C for 80% strain	8 hot-rolling cycles at 550 °C for 240% strain

**Table 2:** Average grain size, nanoparticle size, and density in nanostructured alloys including 14YWT, ODS-Eurofer 97, and HR-Series Alloys examined in this study.

Material	Average Grain Size (µm)	Fine Particle Diameter (nm)	Fine Particle Density (m^−3^)	Ref.
HR-1CC	1.1	39	7.2 × 10^19^	This work
HR-2CC	0.6	23	7.6 × 10^20^	This work
HR-3A	0.8	27	1.5 × 10^19^	This work
ODS-Eurofer 97	2.6	7	1.6 × 10^22^	[40]
14YWT	0.2	3	3.6 × 10^23^	[28]

## Data Availability

The raw data supporting the conclusions of this article will be made available by the authors on request.

## Abbreviations

ADF	annular dark-field
AGI	average grain intercept
APT	atom probe tomography
BF	bright-field
CNA	castable nanostructured alloy
CTMP	continuous thermomechanical processing
DSA	dynamic strain aging
EBSD	electron backscatter diffraction
EDS	energy dispersive X-ray spectroscopy
FIB	focused ion-beam
FM	ferritic/martensitic
HIP	hot isostatic pressing
NDS	nitride dispersion strengthened
NFA	nanostructured ferritic alloy
ODS	oxide-dispersion strengthened
SD	standard deviation
SEM	scanning electron microscopy
SPD	severe plastic deformation
STEM	scanning transmission electron microscopy
TE	total elongation
TEM	transmission electron microscopy
TKD	transmission Kikuchi diffraction
UE	uniform elongation
UTS	ultimate tensile strength
YS	yield stress
